# Visualized Gene Network Reveals the Novel Target Transcripts Sox2 and Pax6 of Neuronal Development in Trans-Placental Exposure to Bisphenol A

**DOI:** 10.1371/journal.pone.0100576

**Published:** 2014-07-22

**Authors:** Chung-Wei Yang, Wei-Chun Chou, Kuan-Hsueh Chen, An-Lin Cheng, I-Fang Mao, How-Ran Chao, Chun-Yu Chuang

**Affiliations:** 1 Department of Biomedical Engineering and Environmental Sciences, National Tsing Hua University, Hsinchu, Taiwan; 2 Schools of Nursing and Health Studies, University of Missouri-Kansas City, Kansas City, Kansas, United States of America; 3 School of Occupational Safety and Health, Chung Shan Medical University, Taichung, Taiwan; 4 Emerging Compounds Research Center, Department of Environmental Science and Engineering, National Pingtung University of Science and Technology, Pingtung County, Taiwan; Florida State University, United States of America

## Abstract

**Background:**

Bisphenol A (BPA) is a ubiquitous endocrine disrupting chemical in our daily life, and its health effect in response to prenatal exposure is still controversial. Early-life BPA exposure may impact brain development and contribute to childhood neurological disorders. The aim of the present study was to investigate molecular target genes of neuronal development in trans-placental exposure to BPA.

**Methodology:**

A meta-analysis of three public microarray datasets was performed to screen for differentially expressed genes (DEGs) in exposure to BPA. The candidate genes of neuronal development were identified from gene ontology analysis in a reconstructed neuronal sub-network, and their gene expressions were determined using real-time PCR in 20 umbilical cord blood samples dichotomized into high and low BPA level groups upon the median 16.8 nM.

**Principal Findings:**

Among 36 neuronal transcripts sorted from DAVID ontology clusters of 457 DEGs using the analysis of Bioconductor limma package, we found two neuronal genes, sex determining region Y-box 2 (Sox2) and paired box 6 (Pax6), had preferentially down-regulated expression (Bonferroni correction p-value <10^−4^ and log2-transformed fold change ≤−1.2) in response to BPA exposure. Fetal cord blood samples had the obviously attenuated gene expression of Sox2 and Pax6 in high BPA group referred to low BPA group. Visualized gene network of Cytoscape analysis showed that Sox2 and Pax6 which were contributed to neural precursor cell proliferation and neuronal differentiation might be down-regulated through sonic hedgehog (Shh), vascular endothelial growth factor A (VEGFA) and Notch signaling.

**Conclusions:**

These results indicated that trans-placental BPA exposure down-regulated gene expression of Sox2 and Pax6 potentially underlying the adverse effect on childhood neuronal development.

## Introduction

Bisphenol A (BPA) has a frequent industrial use as a sealant or flux of plastic materials. Its application results in human exposure through the intake of foods and liquids in polycarbonate water bottles, food wraps, plastic bags, baby bottles, protective coatings on food containers, epoxy resin, and dental composites [1]. BPA is an endocrine-disrupting chemical that mimics hormones through estrogen or thyroid receptor mechanisms, and consequently causes adverse health effects on growth development in an intact organism and its progeny [2], [3]. While some awareness exists about the possible adverse effects of BPA exposure, the broader knowledge of its effects on childhood neuronal development is still limited.

In mammalians, BPA can easily pass through placenta during pregnancy and has a latent effect on postnatal reproductive functions [4]. The reproductive toxicity of BPA is not as high as other environmental chemicals such as 2,3,7,8-Tetrachlorodibenzodioxin (TCDD) or nonylphenol (NP) [5]. However, maternal BPA exposure can cause metabolic and emotional disruption on offspring even in low dose exposure [6], [7]. Urinary BPA has been detected in children in many developed countries e.g., Australia, United States, and Italy [8]–[10]. Our previous bio-monitoring study in Taiwan identified that prenatal BPA exposure concentration is negatively correlated with birth weight and affects gene expression of leptin and adiponectin in male neonates [11]. BPA also causes epigenetic disruptions. Low-dose prenatal BPA exposure alters mRNA expression of epigenetic regulators DNA methyltransferase (DNMT) 1 and DNMT3A in the brain [12]. Rat model indicated that perinatal BPA exposure is a potential causative agent of molar incisor hypomineralization (MIH) during a specific developmental time window [13].

Concerns have been raised about the effect of trans-placental BPA exposure on central nervous system and neuronal development. Early life BPA exposure has been associated with behavior problem such as anxiety, depression, and hyperactivity in children [14]. BPA has been reported in response to childhood behavioral and learning development at age 8–11 [15]. BPA (250 ng/kg/day) enhances fear memory and increases serotonin metabolites 5-hydroxyindoleacetic acid (5-HIAA) levels and 5-HIAA/serotonin (5-HT) in the hippocampus, striatum and midbrain in juvenile female mice [16], and delays perinatal chloride shift by significantly decreasing potassium chloride co-transporter 2 (Kcc2) mRNA expression in developing rat, mouse, and human cortical neurons [17]. BPA also causes adverse effects on neuronal morphology and functions as to interrupting neuronal dendritic and synaptic development in cultures of fetal rat hypothalamus cells at 10 and 100 nM [18]. BPA suppresses neurite extension by inhibiting phosphorylation of mitogen activated protein kinase (MAPK) in rat pheochromocytoma PC12 cells differentiated neuronal-like cells [19]. Furthermore, perinatal exposure to BPA causes GABAergic disinhibition and dopaminergic enhancement that is related to abnormal cortical basolateral amygdala synaptic transmission and plasticity; this effect may be responsible for hyperactivity and attention deficit in BPA-rats [20].

Microarray analysis is an effective way to explore possible mechanisms and has been used to study the molecular pathway of reproductive toxicity of BPA exposure in animal models [21]–[23]. However, few studies evaluated childhood neuronal development in exposure to BPA with human data. In human samples, umbilical cord blood is a postpartum placental remnant containing fetal blood which can be used as a surrogate for childhood study [24]. In this study, we used meta-analysis of publicly available microarray datasets to find the neuronal target genes in exposure to BPA, and explored whether trans-placental BPA exposure in mothers would alter gene expression on their progeny in human umbilical cord blood and the potential underlying mechanism from gene network analysis to childhood neuronal development.

## Materials and Methods

### Data Collection and Differentiated Expression Gene Analysis

Three microarray datasets of human cell models in exposure to low-dose BPA were selected from the ArrayExpress database (http://www.ebi.ac.uk/arrayexpress/) (Table 1). Data were analyzed using a Bioconductor package (http://www.bioconductor.org) implemented in R (http://cran.r-project.org), and were filtered by choosing probes having a standard deviation >0.15 over all the samples in the analysis. Two different platform chips (Agilent Whole Human Genome Microarray and Affymetrix HG-U133Plus2.0) were pre-processing using RMA algorithm (http://rmaexpress.bmbolstad.com/) prior to merging each other. We selected genes from all platforms based on the NIH Entrez Gene ID and used the median rank score method with the R package CONOR [25] for cross-platform normalization (Figure S1). The detailed procedure for performing the meta-analysis was provided in the Supporting Information (Information S1). In the analysis of Bioconductor limma package with t-statistic and false discovery rate (FDR) <0.1, differentially expressed genes (DEGs) with fold change greater or less than ±1.2 were identified individually among each BPA exposure groups (1 pM, 100 pM, 10 nM, 1 uM and 10 uM) versus their corresponding controls between high (1 and 10 uM) and low (1 pM, 100 pM and 10 nM) BPA exposure groups. For gene ontology analysis, DEGs were imported into DAVID Bioinformatics Resources 6.7 to identify major function clusters. To investigate the major neuronal transcripts, Bonferroni correction was used as a screening method to account for multiple hypothesis testing. P-value of Bonferroni correction was 1.45*10^−3^ (0.05/36; adjusted p-value  = p/n, where p is p-value, n is total number of neuronal transcripts) in this study. This study defined 10^−4^ as the cutoff p-value to make selected genes more specific. Therefore, the genes with expression values in the quadrant of greater or less than log-transformed fold change ±1.2 and p-value less than 10^−4^ (the absolute value of log10-transformed value >4) were selected for further network construction and pathway prediction.

**Table 1:** Microarray datasets used in the meta-analysis of gene expression in exposure to BPA.

Study	Accession	Control samples (n)	BPA treated samples (n)	Array Platform	BPA concentration
Qin et al., 2012	E-GEOD-35034	1	1	Agilent-028004 SurePrint G3 Human GE 8x60K Microarray	10 nM
Tiesman, 2011	E-GEOD-17624	4	4	Affymetrix GeneChip Human Genome U133 Plus 2.0	1 pM, 100 pM, 10 nM, 1uM
Huang, 2011	E-GEOD-32160	3	4	Affymetrix GeneChip Human Genome U133 Plus 2.0	1 uM, 10 uM

Source: http://www.ebi.ac.uk/arrayexpress/

### Study Subjects and Sample Collection

This study randomly selected 20 umbilical cord blood samples of 157 healthy pregnant women in a previous birth cohort study [11] recruiting from January 2006 and August 2007 at an obstetrics and gynecology clinic in Hsinchu County, Taiwan. All pregnant women provided their written informed consent of genetic research to participate in this study, and the institutional review boards of National Tsing Hua University and Changhua Christian Hospital approved the bio-sampling process. When pregnant women gave birth, umbilical cord blood samples were collected in glass heparin tubes and delivered at 4°C to the lab within two days for RNA isolation. Plastics were excluded throughout the entire procedure to avoid BPA contamination. Twenty umbilical cord blood samples were dichotomized into high and low BPA level groups based on the median level of BPA exposure (16.8 nM) for determining gene expression of Sox2 and Pax6.

### BPA Detection

The umbilical cord blood samples were centrifuged at 12,000 rpm for 10 min to separate the plasma and corpuscles, and stored at −80°C until analysis. Plasma fraction (500 µl) was mixed with 100 µl of 0.01 M ammonium acetate buffer (pH 4.5; Riedel-de Haen, Seelze, Germany), 4 ml mixture of n-hexane (HPLC grade; Echo Chemical, Miaoli, Taiwan) and diethyl ether (70∶30 v/v, anhydrous; J.T. Baker, Phillipsburg, NJ). Then 8.71 µl of 9.187 M perchloric acid (purity 60–62%; Sigma-Aldrich, St. Louis, MO) was added into the plasma mixture and centrifuged with 3,000 rpm for 5 minutes. After centrifugation, the organic layer was evaporated to dryness and reconstituted with 100 µl of mobile phase (methanol:water 80∶20 v/v) for BPA determination by a reverse-phase high performance liquid chromatography (HPLC) (D-7000) connected to a UV detector (L-7400) consisting of an autosampler (L-7200), a pump (L-2130) and a degasys (DG-2410) (Hitachi High Technologies America, Pleasanton, CA). The QA/QC materials were prepared from a plasma pool in analysis with standard, reagent blank and unknown samples. We performed external calibration using the chromatographic responses of seven standard concentrations in their corresponding solvent. The recovery rates of blanks extended from 96–103%. The relative standard deviation (RSD) among triplicate analyses were 1.99–7.53%, and the recovery percentage was 96.1% with an RSD of 7.53%. The limit of detection (LOD) was 1.75 nM.

### Quantitative Real-Time PCR

The buffy coat of umbilical cord blood sample was pretreated using RBC lysis buffer to avoid the interference of red blood cells. RNA isolation was conducted using Trizol reagent with chloroform and isopropanol. The RNA pellet was washed using 75% alcohol and air dried. Total RNA was dissolved in DEPC contained water and stored under −80°C. Total RNA was reverse-transcribed to cDNA using the high capacity cDNA reverse transcription kits (ABI Inc., Foster, CA) for quantitative real-time analysis. PCR primers (Sox2: F-5′CAC ACT GCC CCT CTC ACA CA3′, R-5′CCC ATT TCC CTC GTT TTT CTT3′; Pax6: F-5′TCG GGC ACC ACT TCA ACA3′, R-5′CGG GAA CTT GAA CTG GAA CTG3′) were designed using Primer Express V.3.0 software (ABI Inc, Foster, CA) according to the mRNA sequence from GenBank. Real-time PCR was performed with the FastStart SYBR Green Master (Roche Inc, Penzberg, Germany) and was analyzed using the 7300 Real-Time PCR system (ABI Inc, Foster, CA). The relative level of mRNA expression was analyzed using comparative method by SDS 1.4 software normalized to the endogenous housekeeping gene β-actin.

### Network Construction and Pathway Prediction

The plug-in system “ClueGO+CluePedia” in the latest version of Cytoscape Software 3.0.2 was used to identify networks and functional pathways of DEGs in response to BPA exposure. ClueGO [26] performs an extensive database of functional interactions including GO, KEGG and Reactome. ClueGO creates the first binary gene-term matrix with the selected terms and their associated genes. Based on this matrix, a term-term similarity matrix is calculated using chance corrected kappa statistics to determine the association strength between the terms. For biological networks, CluePedia [27] calculates the correlation for DEGs based on four tests, Pearson correlation, Spearman's rank, distance correlation and maximal information coefficient (MIC), for investigating linear and non-linear dependencies between implemented variables. In this study, two-sided hypergeometric statistic was performed with kappa score threshold setting of 0.3. Enrichment/depletion was calculated based on Benjamini-Hochberg corrected p-value. GO groups with corrected p-value <0.05 were denoted for different significant levels.

## Results

### Identification of Candidate Genes Relevant to Neuronal Development in Exposure to BPA

**Figure 1:**
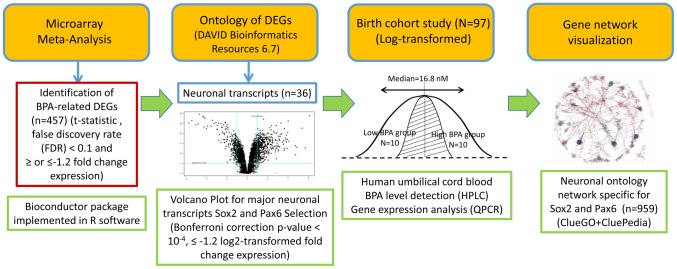
Schematic flow chart of study design. This study processed the meta-analysis of three microarray datasets to identify DEGs in exposure to various BPA levels compared to controls (n = 457). There were 36 neuronal transcripts sorted from 457 DEGs involved in ontology clusters, and top two down-regulated neuronal genes Sox2 and Pax6 were selected in response to BPA exposure. Gene expression of Sox2 and Pax6 were determined in 20 human umbilical cord blood samples randomly recruited from a previous birth cohort [11], and obviously attenuated in high BPA exposure group referred to low BPA group. The visualized gene network of Sox2 and Pax6 and their potential interaction genes specific for neuronal development was predicted in response to trans-placental BPA exposure. DEG: differentially expressed gene; Sox2: sex determining region Y-box 2; Pax6: paired box 6.

**Figure 2:**
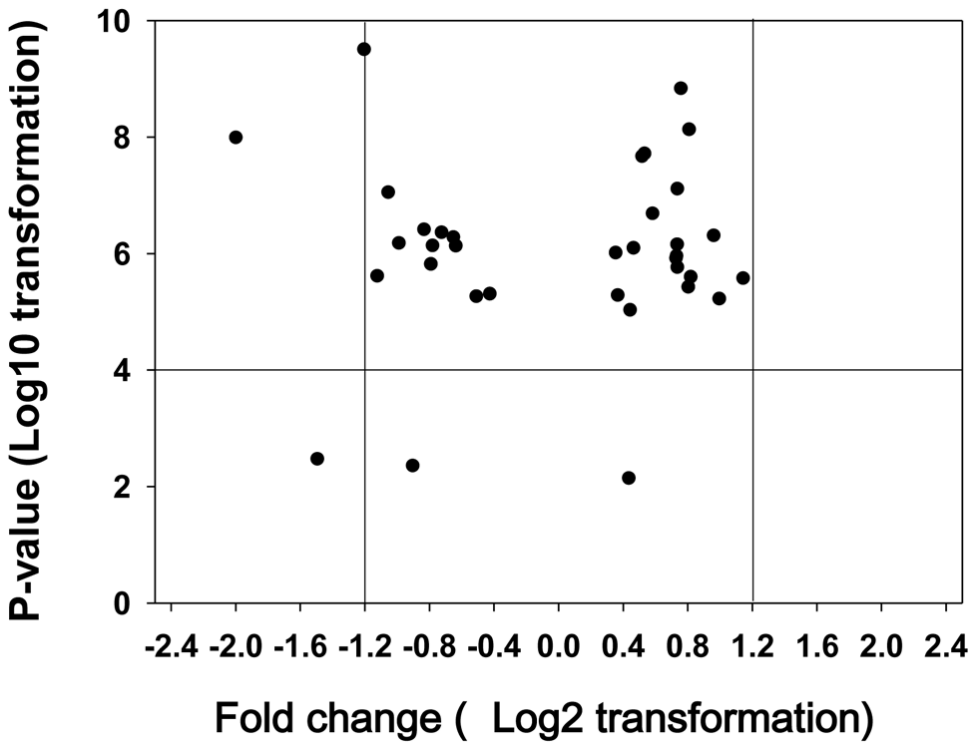
Volcano plot of gene expression relevant to neuronal development in exposure to BPA. X axis is log2-transformed fold change value of gene expression; Y axis is the absolute value of log10-transformed p-value. The genes Sox2 and Pax6 with log-transformed fold change less than −1.2 and p-value less than 10^−4^ were selected as the candidate target genes of neuronal development in trans-placental exposure to BPA.

Figure 1 showed the framework in this study to explore candidate genes relevant to neuronal development in exposure to BPA exposure. This study combined three normalized public microarray datasets (Table 1) and analyzed using Bioconductor limma package to identify 457 DEGs (Table S1) among BPA exposure groups and their controls (1 pM n = 25, 100 pM n = 109, 10 nM n = 145, 1 uM n = 250, 10 uM n = 345). In the DAVID ontology analysis of 457 DEGs, 36 transcripts were relevant to neuronal ontology and their up- and down-regulated expression were listed in Table 2. For exploring candidate genes of neuronal development in response to BPA exposure, 36 potential transcripts relevant to neuronal ontology were illustrated in a volcano plot (Figure 2). Two candidate genes, sex determining region Y-box 2 (Sox2) and paired box 6 (Pax6), had aberrant expression (Bonferroni correction p-value <10^−4^ and ≤−1.2 log2-transformed fold change) in comparison with control respectively responsible for developing neural tube cells and regulating neurogenesis in radial-glial-like neural stem cells [28]. Therefore, Sox2 and Pax6 were served as candidate neuronal genes for further investigation in human umbilical cord blood.

**Table 2:** Significantly 36 down-regulated and up-regulated genes of neuronal ontology in exposure to BPA.

Down-regulated genes				Up-regulated genes			
ID	Name	Fold change (log2 transformed)	p-value	ID	Name	Fold change (log2 transformed)	p-value
PAX6	paired box 6	−1.9937	1.05E–08	ESR1	estrogen receptor 1	1.1477	2.74E–06
UNC5B	unc-5 Homolog B	−1.4892	3.45E–06	ATP1A2	ATPase, Na+/K+ transporting, alpha 2 polypeptide	0.9995	6.13E–06
SOX2	SRY (Sex Determining Region Y)-Box 2	−1.20	3.22E–10	TGFB2	transforming growth factor, beta 2	0.9657	5.07E–07
PHGDH	phosphoglycerate Dehydrogenase	−1.1190	2.50E–06	TUBB2A	tubulin, beta 2A class IIa	0.8229	2.58E–06
MIB1	mindbomb E3 ubiquitin protein ligase 1	−1.0507	9.18E–08	BAIAP2	BAI1-associated protein 2	0.8133	7.60E–09
EIF2AK3	eukaryotic translation initiation factor 2-alpha kinase 3	−0.9850	6.85E–07	SEMA3A	sema domain, (semaphorin) 3A	0.8079	3.86E–06
KLHL24	Kelch-Like Family Member 24	−0.8977	0.004542	PLXNB2	plexin B2	0.7616	1.51E–09
TBCE	tubulin folding cofactor E	−0.8284	3.98E–07	FGFR1	fibroblast growth factor receptor 1	0.7410	1.77E–06
JMJD6	jumonji domain containing 6	−0.7862	1.56E–06	TUBB3	tubulin, beta 3 class III	0.7405	7.92E–08
NF1	Neurofibromin 1	−0.7749	7.51E–07	SLITRK5	SLIT and NTRK-like family, member 5	0.7387	7.19E–07
RUFY3	RUN and FYVE domain containing 3	−0.7190	4.47E–07	NCS1	neuronal calcium sensor 1	0.7352	1.13E–06
PPM1A	protein phosphatase, mg2+/mn2+ dependent, 1a	−0.6465	5.42E–07	ZNF488	zinc finger protein 488	0.7329	1.24E–06
PEX13	peroxisomal biogenesis factor 13	−0.6320	7.58E–07	BMP4	bone morphogenetic protein 4	0.5869	2.12E–07
DLC1	deleted in liver cancer 1	−0.5047	5.61E–06	ERBB2	v-erb-b2 avian erythroblastic leukemia viral oncogene homolog 2	0.5367	1.98E–08
GSK3B	glycogen synthase kinase 3 beta	−0.4210	5.05E–06	JAG2	jagged 2	0.5218	2.21E–08
PPARA	peroxisome proliferator-activated receptor alpha	−0.2660	5.24E–06	EPHA7	EPH receptor A7	0.4680	8.24E–07
				LRP8	low density lipoprotein receptor-related protein 8, apolipoprotein e receptor	0.4476	9.53E–06
				ADORA1	adenosine A1 receptor	0.4396	0.007408
				IQCB1	IQ motif containing B1	0.3704	5.36E–06
				EPHB2	EPH receptor B2	0.3587	9.93E–07

### Comparison of Gene Expression between High and Low BPA Levels in Cord Blood Samples

**Figure 3:**
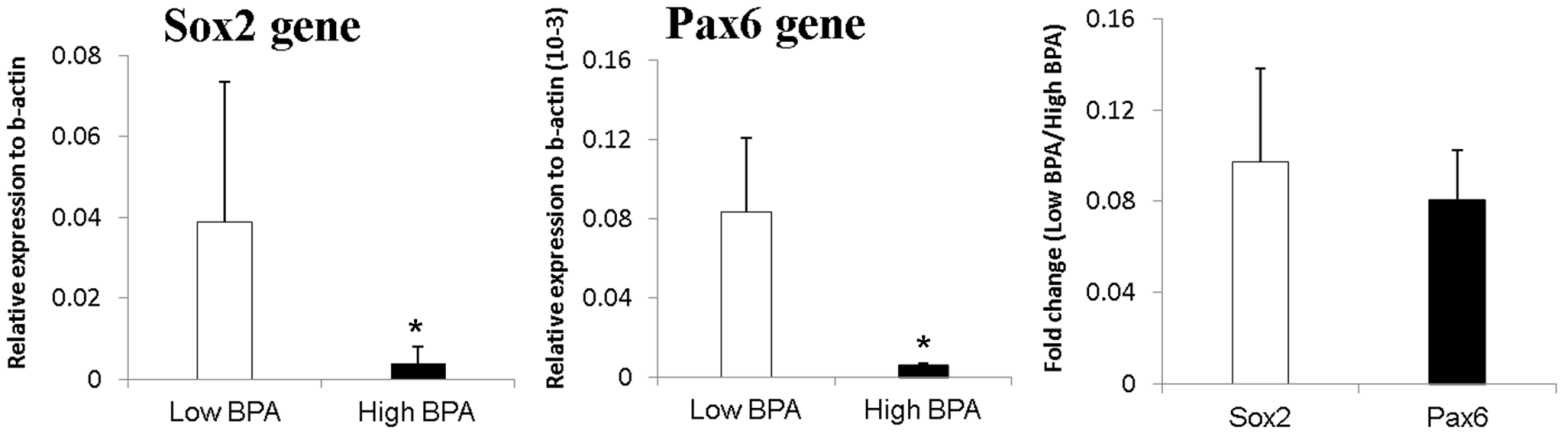
Gene expression and fold change of Sox2 and Pax6 between high and low BPA exposure groups in human umbilical cord blood samples. Both Sox2 and Pax6 were significantly down-regulated in high BPA exposure group. The expression of Sox2 and Pax6 were relative to house-keeping gene b-actin.

Twenty human umbilical cord blood samples were randomly selected from our previous birth cohort [11] and their average BPA level were 23.6 nM. The gene expression of Sox2 and Pax6 were determined in low BPA group and high BPA group classified upon the median value (16.8 nM) of BPA in umbilical cord blood. Both gene expression of Sox2 and Pax6 significantly decreased in higher BPA group referred to lower BPA group (fold change 0.1 and 0.08, respectively) (Figure 3). Results suggested that trans-placental BPA exposure might affect childhood neuronal development underlying decreased Sox2 and Pax6 expression.

### Neuronal Network Reconstruction with Sox2 and Pax6

**Figure 4:**
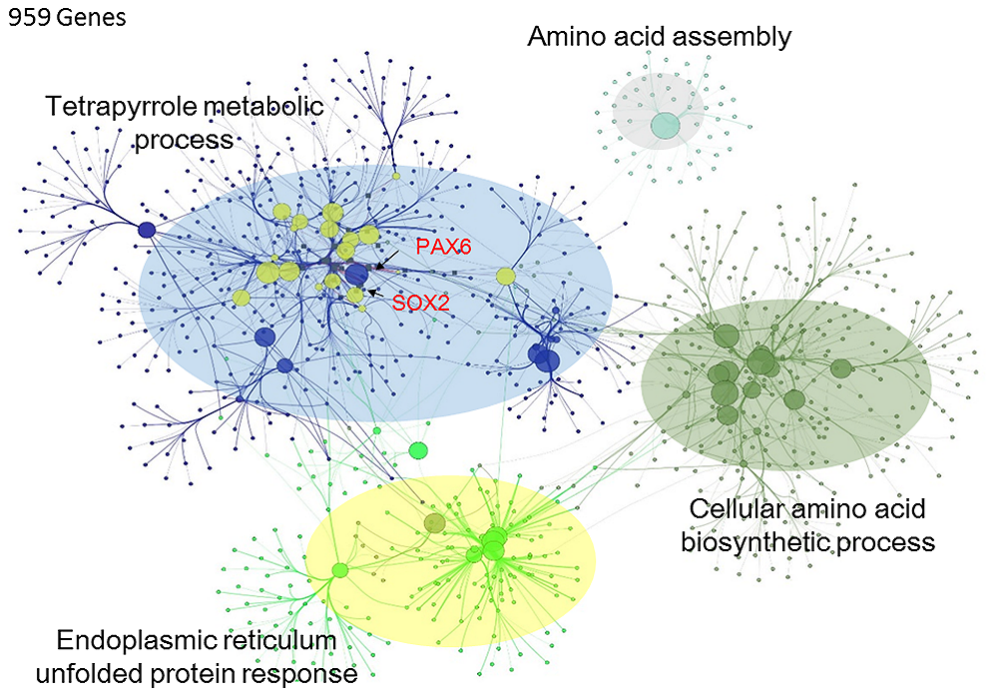
Gene regulatory network and gene ontology in exposure to BPA. Totally 959 genes connecting with the 457 DEGs relevant to BPA exposure constructed this gene network. The gene ontology enrichment of the 959 genes mapped for GO category presented in tetrapyrrole metabolic process (Sox2 and Pax6 were involved in), amino acid assembly, endoplasmic reticulum unfolded protein response, and cellular amino acid biosynthetic process.

**Figure 5:**
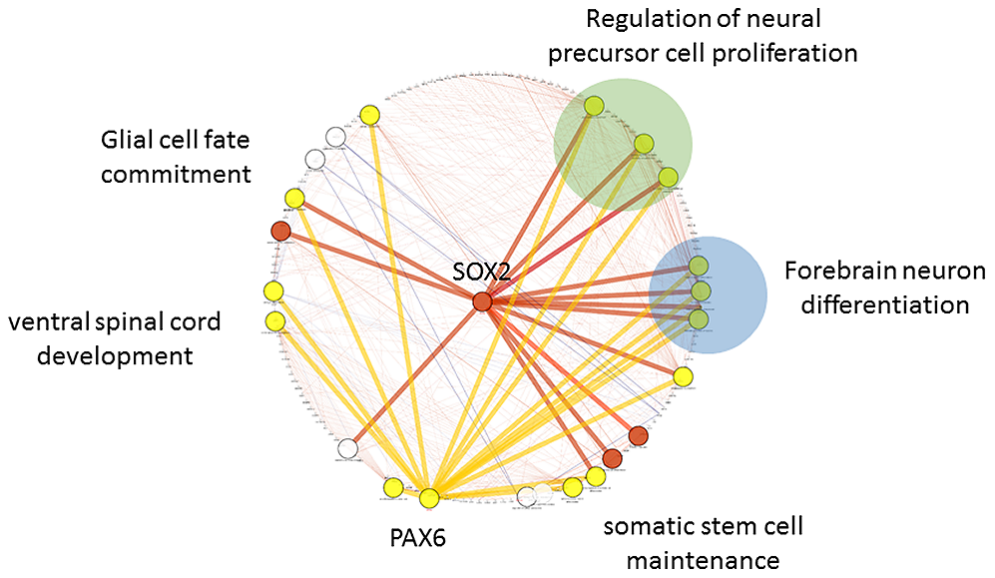
Sub-network illustration specific to Sox2 and Pax6 as hub genes. The visualized sub-network illustrated in the specific neuronal functions for Sox2 and Pax6 genes. Sox2 and Pax6 had similar neuronal functions such as regulation of neural precursor cell proliferation and forebrain neuron differentiation.

This study imported 457 DEGs into Cytoscape plug-in ClueGO+CluePedia to investigate the gene network and functional pathway prediction in response to BPA exposure. The results of Cytoscape analysis presented that totally 959 genes constructed a gene network connecting with the 457 DEGs (Figure 4). The gene ontology enrichment of the 959 genes relevant to BPA exposure mapped for GO category presented in tetrapyrrole metabolic process, cellular amino acid biosynthetic process, endoplasmic reticulum unfolded protein response, and amino acid assembly. The further sub-network analysis in Figure 5 was visualized specifically to understand the neuronal functions that Sox2 and Pax6 were involved. Sox2 and Pax6 had similar neuronal functions such as regulation of neural precursor cell proliferation and forebrain neuron differentiation.

**Figure 6:**
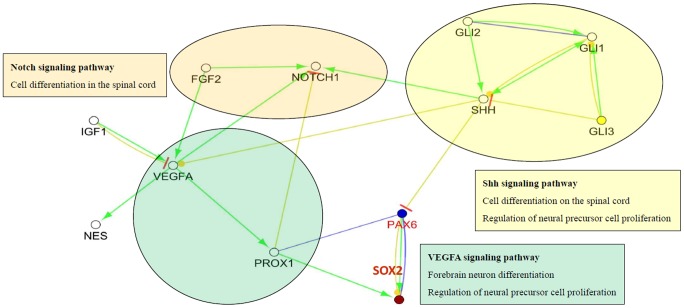
A predicted pathway of Sox2 and Pax6 and their potential interaction genes resulted from BPA exposure. Sox2 and Pax6 acted in Shh, Notch and VEGFA pathway for cell differentiation of spinal cord, forebrain neuron differentiation, and regulation of neural precursor cell proliferation. In Shh signaling, Shh is positively regulated by transcription factor encoded genes Gli1 and Gli2 and negatively modulated by Gli3. In VEGFA pathway, VEGFA generally activates Prox1 that up-regulated Sox2 and Pax6, and suppresses Notch1. Notch signaling indirectly modulates Sox2 and Pax6 through Shh, VEGFA and Prox1. In exposure to BPA, Shh down-regulated Sox2 through Pax6 antagonism, and IGF1 attenuated VEGFA expression and its down-stream genes. Shh: Sonic hedgehog; VEGFA: vascular endothelial growth factor A; IGF1: insulin-like growth factor 1; Green line: activation; blue line: binding; yellow line: expression; yellow line with a red bar: antagonizing.

The results of ClueGO analysis presented a predicted pathway of Sox2 and Pax6 and their potential interaction genes resulted from BPA exposure in Figure 6. Sox2 and Pax6 acted in sonic hedgehog (Shh), Notch and vascular endothelial growth factor A (VEGFA) pathway for cell differentiation of spinal cord, forebrain neuron differentiation, and regulation of neural precursor cell proliferation. In Shh signaling, Shh is positively regulated by transcription factor encoded genes Gli1 and Gli2 and negatively modulated by Gli3. Regarding to BPA exposure, Shh down-regulates Sox2 through Pax6 antagonism. In VEGFA pathway, VEGFA generally activates prospero-related homeobox 1 (Prox1) to up-regulate Sox2 and Pax6, and suppresses Notch1. BPA exposure would cause insulin-like growth factor 1 (IGF1) to attenuate VEGFA expression and its down-stream genes. Notch signaling indirectly modulates Sox2 and Pax6 through Shh, VEGFA and Prox1 in response to BPA exposure.

## Discussion

Human exposure to chemicals relevant to disease outcome is difficult to be determined and estimated, especially for the effect on child development. Although some studies have found that BPA affects growth and development of reproductive organ [29], [30], potential adverse effects of BPA on childhood neuronal development are not fully understood yet. In this study, we identified Sox2 and Pax6 as neuronal development biomarkers whose gene expression was appeared in response to trans-placental BPA exposure. Such a biomarker holds promise in assessing BPA exposure and acts as a clinically relevant predictor for neurogenesis in children underlying maternal BPA exposure. In general, it's hard to explore the health effect of prenatal exposure in human subjects. A biomarker is found from a costly method because of the need for several gene chips with sufficient amount of samples; childhood neuronal development research takes time to prospectively follow up a cohort which generates additional challenges. The method described in this study offered an alternative strategy to examine the molecular effect of prenatal BPA exposure on child development. Candidate biomarkers were surveyed from the use of microarray meta-analysis and the gene expression of biomarkers were investigated in human samples from fetal umbilical cord blood for potential impact research.

In this study, the step-wise approach including the meta-analysis of microarray DEGs, GO and Bonferroni correction between high and low BPA exposure was used to investigate the neuronal candidate genes Sox2 and Pax6 in exposure to BPA. Additionally, the gene expression of Sox2 and Pax6 were determined in human fetal cord blood to evaluate whether it could be used as biomarkers for childhood neurogenesis deficiency resulting from trans-placental BPA exposure. This study found higher BPA exposure level in human fetal cord blood samples decreased gene expression of Sox2 and Pax6. The visualized network analysis showed that Sox2 and Pax6 were highly involved in the function of neurogenesis. This alternative method to evaluate the impact of xenobiotics exposure on human is more effective than an animal model, and more convenient and time-saving than a cohort study for a long time follow-up.

Several but not many studies evaluated the impact of low-dose BPA exposure (<100 nM) on neuronal development. In human embryonic stem cells, BPA exposure (1, 10 or 100 nM) influences mammosphere area and down-regulate the expression of E-cadherin protein in the early differentiation stage of mammary epithelial cells [31]. In rattus experiment, maternal BPA exposure at 0.05, 0.5, 5 or 50 mg/kg per day from embryonic day 9 to day 20 in rats affects fetal growth (locomotor activity, exploratory habits and emotional behavior in open field test), synaptic structure (widened synaptic cleft, thinned postsynaptic density and unclear synaptic surface), and decreases mRNA and protein expressions of synaptophysin, PSD-95 (postsynaptic density protein 95), spinophilin, GluR1 (glutamate receptor 1) and NMDAR1 (N-methyl-D-aspartate receptor 1) in the hippocampus of male offspring on postnatal day 21 [32]. In neuronal or neuronal-like cells, neuronal differentiation is decreased in pheochromocytoma PC12 cells in pre-exposure to BPA (0.1, 1, 10 or 100 nM) for one week, longer or a week followed by a week's withdrawal [33]. PC12 cells treated with 43.8 nM BPA for 5 days suppress neurite extension through inhibition of phosphorylation of mitogen-activated protein kinase [19]. Rats exposed to 10 or 100 nM BPA for 7 days increases both MAP2 (microtubule associated protein-2) and synapsin I-positive areas for neuronal development as well as synaptic densities in hypothalamic neurons and glias [18]. In mouse purified astrocyte and neuron/glia co-cultures, exposure to low-dose BPA (100 fM, 1 pM, 10 pM, 100 pM, 1 nM, 10 nM or 100 nM) activates astrocytes, increases GFAP (glial fibrillary acidic protein) level and enhances Ca2+ responses to dopamine, which may contribute to potentiate the development of psychological dependence on supersensitivity to psychostimulant-induced pharmacological actions such as drugs of abuse [34]. Additionally, here are some BPA level studies in other countries for references. Serum BPA level of pregnant women (n = 61), fetuses (n = 61) and non-pregnant women (n = 26) in Eastern Townships of Canada ranged from non-detectable (ND) to 19.6 nM, ND to 20.2 nM, and 5.7 to 35.9 nM, respectively [35]. The cord blood BPA level of 106 boys in France was from 0.6 to 20.9 nM [36]. Also, BPA level in maternal blood and cord blood (n = 300) in Korea was from ND to 292.5 nM and ND to 38.9 nM, respectively [37]. These data supported that the measurement of BPA level in this study is similar with the range in other countries.

Prenatal BPA exposure impairs murine fetal neocortical development by accelerating neuronal differentiation/migration during the early embryonic stage to affect neuronal plasticity and interferes corticosterone and corticosteroid receptors in the hippocampus [35]–[37]. According to mother report or behavior assessment system for children 2 (BASC-2) and behavior rating inventory of executive function-preschool (BRIEF-P), prenatal BPA exposure is significantly concerned in childhood behavioral problems such as anxiety, depression, inattention and hyperactivity in children less than age 7 [38], [39]. However, these findings didn't further explore momentous biomarkers of neurogenesis relevant to trans-placental BPA exposure. The network analysis of this study presented that Sox2 and Pax6 were mainly involved in the regulation of neural precursor cell proliferation and forebrain neuron differentiation. A study using human embryonic stem cell-derived NPC for neurogenesis modeling found that Sox2 is required to maintain optimal levels of lin-28 homolog A (LIN28), a well-characterized suppressor of lethal-7 (let-7) microRNA biogenesis, and Sox2 loss causes proliferation deficit and differentiation inhibition in NPCs because of the abnormal expression of LIN28 and let-7 [40]. Sox2 is highly expressed in undifferentiated cells and declines with differentiation, and knockdown of Sox2 expression makes beta-tubulin-positive cells fail to progress to more mature neurons [41]. Aberrant expression of Sox2 is also related to childhood neuronal development. A study recruited 10 patients aged from 2 to 9 years screening for gene mutation found that Sox2 mutation is genetically correlated with inherited ocular phenotypes, anophthalmia and microphthalmia [42]. A review report presented that mutation of several transcription factors, including Sox2 gene, results in congenital hypopituitarism or septo-optic dysplasia in murine neonates [43]. Additionally, Pax6 knockdown reduces neurogenic capacity in embryonic stem cells [44]. Constitutive activation of Wnt signaling pathway in forebrain or brainstem precursor cells causes dramatic brain enlargement as well as in the formation of medulloblastoma, a malignant brain tumor in children [45]. Activated Wnt signaling leads to a virtually loss of Pax6 expression, and causes disruption in the proliferation and migration of neurons in mice [46]. Pax6 mutation affects downstream genes on mice cerebellar development for disturbed survival and migration and defects of neurite extension in producing granule cells [47]. Two case reports individually mentioned that mutated Pax6 results in reduced vision, photophobia and eyelid ptosis in an autistic child patient, and is responsible for impaired auditory sensory and higher order interhemispheric transfer in a 12 year old child [48], [49]. Above studies indicated that Sox2 and Pax6 are relevant to childhood neurogenesis. In our study, we identified that BPA exposure affects Sox2 and Pax6 prenatally, and this might cause adverse effects on aberrant neuronal or behavioral development for children along with their increasing age.

This study found that BPA exposure decreased the gene expression of Sox2 and Pax6 in fetal cord blood, and Sox2 and Pax6 were involved in neuronal development according to network analysis. Findings in this study were consistent with a Xenopus laevis model that prenatal BPA exposure decreases the expression of Sox2 and Pax6 and disrupts Notch signaling to inhibit gamma-secretase activity for neurodegenerative abnormalities [50]. BPA also decreases the expression of Pax6, but not Sox2 to cause malformation of the head region in embryos through estrogen receptor 1 (ESR-1) and Notch signaling in Xenopus laevis [51]. Indirect evidence showed that decreased expressions of Sox2 and Pax6 cause adverse effects on neuronal development. Sox2 and Pax6 are lost and undergo glial differentiation after 5-bromo-2′-deoxyuridine (BrdU) exposure [52]. Conditionally, deleted Sox2 and Pax6 cause proliferative defects, alter morphology and reduce clonogenicity in forebrain-derived neural stem cells [28]. The results of fetal cord blood and network analysis in this study also supported that BPA exposure down-regulated Sox2 and Pax6 expression.

The visualized network analysis in response to BPA exposure was addressed in Figure 6 that illustrated that Shh, Notch and VEGFA pathways were in regulation to Sox2 and Pax6 for neuronal signaling including cell differentiation of spinal cord, forebrain neuron differentiation, and regulation of neural precursor cell proliferation. In exposure to BPA, Sox2 and Pax6 were down-regulated by Shh signaling and IGF1 attenuating VEGFA. Pax6 regulates the proliferation of neural progenitor cells in cortical subventricular zone through direct modulation of the Sox2 expression during the late developmental stage in mice [53]. Meis homeobox 2 (Meis2) activates Sox2 through the up-regulation of Pax6 for lens epithelial cell differentiation [54]. In human autopsy samples, the expressions of Sox2 and Pax6 are both higher in the radial glial cells and intermediate progenitors in the third trimester preterm birth, an important period for neurogenesis [55].

Shh pathway plays a role in many processes during embryonic development and remains active in the adult involving in the maintenance of stem cells [56]. Shh supports survival and stimulates growth of motor neurons, neurite outgrowth and neurosphere formation in murine primary embryonic spinal cord cell culture [57]. Shh pathway containing three cubitus interruptus (Ci) homologues, Gli1, Gli2 and Gli3, mediates Hedgehog-dependent cell fate specification in the developing spinal cord. The majority of slow-cycling neural stem cells (NSCs) express Gli2 and Gli3, whereas Gli1 is restricted ventrally, and all three genes are down-regulated when NSCs transition into proliferating progenitors [58]. Multiple ventral neuronal types can develop in the absence of Gli function in mice and chick embryos developing spinal cord models, but require balanced Gli protein activities for their correct patterning and differentiation [59]. Additionally, Shh/Gli1signaling is up-regulated in both mRNA and protein levels of the malignant glioma cells in U87-implanted nude mice, and down-regulated after curcumin treatment [60]. Shh signaling is activated accompanying with higher expression of miR-183∼96∼182 for medulloblastomas development in mice [61]. Taken above studies together, it suggested that BPA exposure might activate Shh to down-regulate Sox2 and Pax6 potentially for glioma and medulloblastoma brain cancers. However, prenatal exposure to BPA in mice produced significant decrease in the dopaminergic neuron development factors, Shh protein and glial cell line-derived neurotrophic factor [62], which is controversial to the prediction in this study after BPA exposure.

In general, VEGFA positively regulates Sox2 through Prox1 gene in embryonic eye development and lens differentiation [63]. VEGFA is required for normal forebrain development and downstream consequences for NSC fate decisions [64]. In this study, we proposed the induced expression of IGF1 to attenuate VEGFA in response to BPA exposure that led to down-regulated Sox2 and Pax6. IGF1 has been identified as a crucial factor in the central nervous system and is involved in cognitive functions, brain aging and development [65]. While circulating blood IGF1 and IGF2 levels exert trophic effects on neurogenesis and neuronal survival, CNS-derived or intrathecally derived IGF1 is also important in maintaining normal brain function [66]. IGF1 has been found to inhibit axon regeneration in aging C. elegans motor neurons [67]. The DNA methylation pattern of IGF1 and the ability of NSC differentiation are inhibited from alcohol consumption in human NSCs that means over-expression of IGF1 is harmful to neuronal development [68]. In an oxygen-induced retinopathy mice model, knockout of insulin or IGF1 receptor was associated with blunted elevation of VEGF, endothelial nitric oxide synthase and endothelin-1. According to these findings, we inferred that BPA exposure up-regulates IGF1 to antagonize VEGFA and down-regulates Sox2 through Prox1 gene that would be baneful to normal neuronal development and differentiation.

Notch pathway has been recognized as one of the main contributors in regulating neural development and has been proposed as a key mediator in neuroplasticity [69]. Notch intervenes with gp130 pathway at many stages to determine cell fate from the first neural lineage commitment and generation of neuronal precursors for the terminal specification of cells as neurons and glia [70]. In temporal lobe epilepsy patients, Notch signaling is up-regulated in response to epileptic seizure activity, and its activation further promotes neuronal excitation of CA1 pyramidal neurons in acute seizures [71]. In the developing chicken spinal cord, a disintegrin and metalloprotease 10 (ADAM10) negatively regulated Notch1 to increase the number of beta-III-tubulin-positive cells during neural progenitor cell differentiation [72]. Some studies started to focus on the pathway interactions. Shh mediated up-regulation of Notch1 is attenuated after cyclopamine treatment in both bovine retinal endothelial cells (BRECs) and pericytes (BRPs). As to anti-angiogenesis in cancer therapy, Notch signaling functions in the physiologic response to loss of VEGF signaling, and thus participates in tumor adaptation to VEGF inhibitors in human NGP neuroblastoma cells [73]. According to these findings, we addressed two explanations for Notch signaling on Sox2 and Pax6 expression in exposure to BPA. Notch1 down-regulates Sox2 and Pax6 in the process of Shh or VEGFA activation. Additionally, Notch1 was inhibited by VEGFA-activated Prox1 playing a feedback role for Sox2 and Pax6 expression. Based on above evidence, this study suggested BPA exposure would suppress neuronal transcripts Sox2 and Pax6 expression in fetal cord blood to cause neuronal development defect underlying the complicated regulations of Shh, VEGFA and Notch signaling pathways.

## Conclusions

This study presented an alternative way to investigate the adverse effect of trans-placental xenobiotics exposure on child development in meta-analysis of public microarray datasets for exploring target genes and their potential gene network of neuronal functions. In the network-analysis of BPA exposure, we identified Sox2 and Pax6 as major neuronal transcript candidates and were involved in tetrapyrrole metabolic process. Sub-network analysis identified that Sox2 and Pax6 were highly involved in regulation of neuronal precursor cells and maintenance of stem cells, which supported the importance of Sox2 and Pax6 in neurogenesis. Furthermore, the decreased expression of Sox2 and Pax6 genes were determined in higher BPA level group of fetal umbilical cord blood. The findings suggested that trans-placental BPA exposure might down-regulate Sox2 and Pax6 expression and cause adverse effect on childhood neuronal development. It is necessary to pay attention in maternal BPA exposure during the period of pregnancy and its effect on child development.

## Supporting Information

Figure S1 **Process of matching probes among different microarray platforms.** Microarrays from Affymetrix and Agilent platforms were originally normalized and summarized using RMA and 75^th^ percentile normalization methods, respectively. Probes were combined to take the average measurements for genes with more than two probes. After the combination of genes from both platforms, correlation and median rank score (MRS) were used to select appropriate number of genes for further investigation.
(TIF)

Table S1 **457 DEGs in response to BPA exposure and their corresponding ontology clusters.**
(DOC)

Information S1 **Description text of microarray data processing and differentially expressed analysis.**
(DOCX)
