# Supplementary figures and images for: Visualized Gene Network Reveals the Novel Target Transcripts Sox2 and Pax6 of Neuronal Development in Trans-Placental Exposure to Bisphenol A
Source: PLoS One. 2014 Jul 22;9(7):e100576. doi: 10.1371/journal.pone.0100576 (PMC4106758; doi:10.1371/journal.pone.0100576)

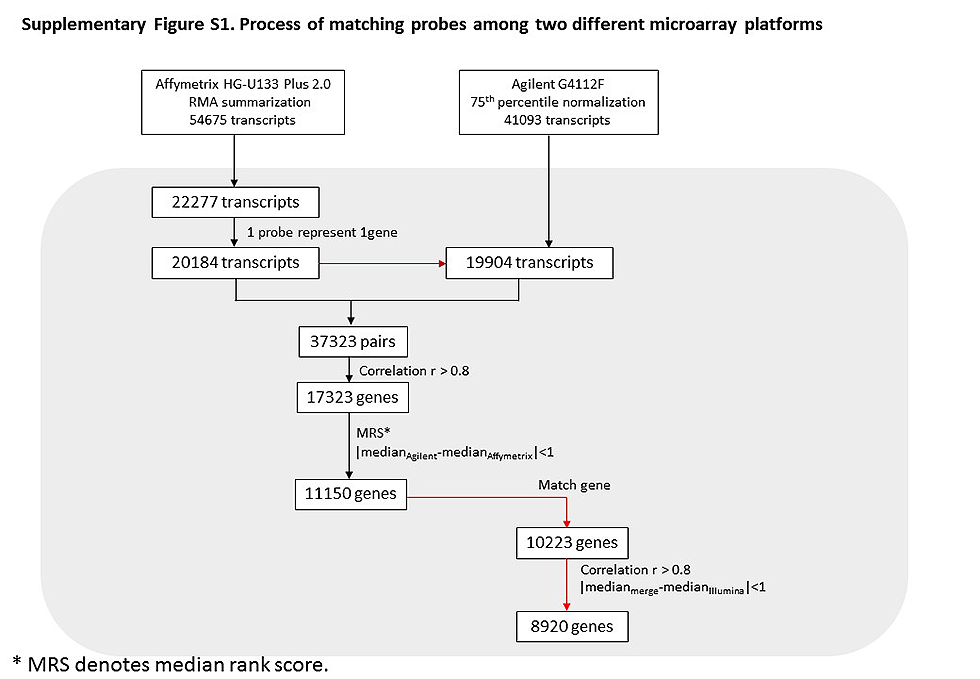

Supplement: Figure S1 — Process of matching probes among different microarray platforms. Microarrays from Affymetrix and Agilent platforms were originally normalized and summarized using RMA and 75th percentile normalization methods, respectively. Probes were combined to take the average measurements for genes with more than two probes. After the combination of genes from both platforms, correlation and median rank score (MRS) were used to select appropriate number of genes for further investigation. (TIF) [file pone.0100576.s001.tif]
