# Supplementary material for: Visualized Gene Network Reveals the Novel Target Transcripts Sox2 and Pax6 of Neuronal Development in Trans-Placental Exposure to Bisphenol A
Source: PLoS One. 2014 Jul 22;9(7):e100576. doi: 10.1371/journal.pone.0100576 (PMC4106758; doi:10.1371/journal.pone.0100576)
Supplement: Table S1 — 457 DEGs in response to BPA exposure and their corresponding ontology clusters. (DOC) [file pone.0100576.s002.doc]

**Table S1. 457 DEGs in response to BPA exposure and their corresponding ontology clusters**

| Ontology Clustering | Genes | Count | P-value |
| --- | --- | --- | --- |
| neuron projection | ATP1A2, EPHA7, FGFR2, RUFY3, ADORA1, ESR1, NCS1, GSK3B, KLHL24, NF1, PPM1A, SEMA3A, TGFB2, TUBB3, JMJD6 | 15 | 5.80E-02 |
| neuron differentiation | BAIAP2, EPHA7, EPHB2, SLITRK5, SOX2, BMP4, FGFR1, JAG2, PAX6, PHGDH, SEMA3A, TGFB2, TBCE, TUBB2A, TUBB3, UNC5B, ERBB2 | 17 | 9.70E-02 |
| regulation of oligodendrocyte differentiation | BMP4, NF1, ZNF488 | 3 | 2.10E-02 |
| negative regulation of cell differentiation | EPHB2, IQCB1, RUFY3, SOX2, BMP4, MIB1, NF1, PAX6, PPARA, SEMA3A | 10 | 1.00E-01 |
| negative regulation of neurogenesis | EPHB2, RUFY3, BMP4, NF1, SEMA3A | 5 | 2.80E-02 |
| regulation of nervous system development | EPHB2, RUFY3, SOX2, BMP4, EIF2AK3, MIB1, NF1, PAX6, PLXNB2, SEMA3A, ZNF488 | 11 | 2.60E-02 |
| regulation of neurogenesis | EPHB2, RUFY3, SOX2, BMP4, MIB1, NF1, PAX6, PLXNB2, SEMA3A, ZNF488 | 10 | 2.70E-02 |
| forebrain development | SOX2, BMP4, DLC1, LRP8, NF1, PAX6, PEX13 | 7 | 1.90E-01 |
| neuron fate commitment | SOX2, BMP4, JAG2, JMJD6, PAX6, TGFB2 | 6 | 2.20E-02 |
| response to endoplasmic reticulum stress | AARS, COL4A3BP, EIF2AK3, ERN1, GSK3B, HERPUD1, HSPA5, PPP1R15B | 8 | 2.00E-05 |
| regulation of hydrolase activity | ACAP2, FGD3, GM2A, PXK, TBC1D15, TBC1D9B, DLC1, ERN1, EIF2AK3, HERPUD1, HSPA5, MOAP1, NF1, PPP1R2, TGFB2, TPM1, TNFSF15, TNFRSF10B | 18 | 6.10E-03 |
| regulation of cell motion | ADORA1, DLC1, IRS2, IGFBP3, JAG2, NF1, PAX6, PTP4A1, SEMA3A, TGFB2, TPM1 | 11 | 2.70E-02 |
| endoplasmic reticulum | AGPAT1, ADORA1, ATL2, BET1, BNIP1, COL4A3BP, CYP2R1, DHCR7, DNAJB9, DNAJC3, EIF2AK3, ERN1, FDFT1, FMO5, GOLT1B, GOSR2, HERPUD1, HSD17B7, HSPA13, HSPA5, LPCAT1, MOSPD2, NPC1, ORMDL1, PDIA4, PIGL, PIGX, PLA2G12A, PREB, PTP4A1, RAB21, SC5DL, SCD, SCFD1, SEC63, SEL1L, SERP1, TMEM173, TMEM38A, TMEM38B, TMEM98, UBE2J1, YIPF5 | 43 | 4.30E-06 |
| amino-acid biosynthesis | AGPAT1, MTRR, UGDH, ASNS, LIAS, PHGDH, PSPH, SCD, SC5DL | 9 | 1.60E-02 |
| apoptosis | ARF6, ARHGEF17, BCL2L15, BNIP1, CASP10, CYFIP2, DLC1, DYRK2, EIF2AK3, ERN1, FAF1, FEM1B, FGD3, FXR1, GADD45A, GLRX2, KLF11, MDM4, MEF2A, MOAP1, PAK2, PDCD5, PPP1R13B, PPP3CC, ROCK1, RYBP, SEMA3A, SIAH2, STK17B, TMEM173, TNFRSF10B, TNFSF15, TRIB3, UNC5B | 34 | 3.10E-05 |
| nucleotide-binding | ARF6, ARL4C, ABCC5, ATP1A2, DDX55, EPHA1, EPHA7, EPHB2, HBS1L, MKNK2, NLRX1, NUAK1, RAB21, SMG1, ACSS2, AARS, ASNS, ATL2, CSNK2A1, CRY1, DYRK2, ERN1, EIF2AK3, FGFR1, FGFR2, GCLC, QRSL1, GSK3B, GNAI3, GNL2, HSPA4, HSPA9, HSPA13, HKDC1, HSPA5, LONP1, MARS, PAK2, PCK2, PIK3CA, PIK3C2A, GART, RHOQ, STK17B, STK36, STK40, SIK3, SARS, ROCK1, SPATA5L1, SRXN1, TRPM7, TIMM44, TRIM23, WARS, TUBB2A, TUBB3, TUBE1, UBE2J1, ERBB2 | 60 | 3.70E-04 |
| actin filament-based process | ARF6, FHDC1, FGD3, ARHGEF17, CYTH2, DLC1, MYL6B, NF1, OBSL1, PAK2, RHOQ, ROCK1, TRPM7, TPM1 | 14 | 9.30E-03 |
| golgi apparatus | ARF6, RAB21, ST6GALNAC6, YIPF5, BICD1, CHST4, COL4A3BP, COG3, ECE2, FNDC3A, NCS1, GOSR1, GOSR2, GOLGA3, GOLGA4, GOLT1B, BET1, LPCAT1, OSBP, PIK3C2A, SCFD1, ROCK1, TRIM23 | 23 | 1.30E-02 |
| glutamine family amino acid metabolic process | ASNS, GCLC, GCLM, GLS, GFPT1, PHGDH | 6 | 1.00E-02 |
| regulation of cell death | BNIP1, EPHA7, FGD3, FAF1, ARHGEF17, SOX4, ADORA1, AARS, ASNS, BMP4, CASP10, DLC1, DYRK2, ERN1, ESR1, EIF2AK3, FEM1B, GCLC, GCLM, GSK3B, HSPA9, HERPUD1, HSPA5, IGFBP3, JAG2, MOAP1, NF1, NME5, PIK3CA, PDCD5, PPP1R13B, PPP3CC, STK17B, ROCK1, TGFB2, TNFSF15, TNFRSF10B, ERBB2 | 38 | 4.90E-04 |
| Zinc finger, RING-type, conserved site | BRAP, CBLB, KIAA0182, LONRF1, MDM4, RBCK1, ARIH1, CCNB1IP1, MKRN2, MIB1, RNF150, RNF41, RNFT1, SIAH2, TRIM23 | 15 | 6.70E-03 |
| regulation of catabolic process | CBLB, FAF1, MDM4, NPC1, ADORA1, ARNTL, GCLC, IRS2, PPARA | 9 | 3.20E-03 |
| intracellular transport | CBLB, GRPEL2, NXT1, NPC1, RPAIN, SEC63, SMG1, ARNTL, ATL2, BLOC1S3, COG3, XPO5, XPOT, FAM160A2, GSK3B, GOSR1, GOSR2, GOLGA3, HSPA9, BET1, KPNA1, MYL6B, PEX13, PREB, SCFD1, SRP72, TOM1L2, TBRG1, TGFB2, TIMM44, TNPO1, TPM1 | 32 | 7.50E-04 |
| Steroid biosynthesis | DHCR7, FDFT1, HSD17B7, LSS, SC5DL | 5 | 9.40E-04 |
| heat shock protein binding | DNAJB9, DNAJC2, DNAJC3, DNAJC7, FAF1, SEC63, ARNTL, ERBB2 | 8 | 2.00E-03 |
| protein folding | DNAJB9, DNAJC2, DNAJC7, GRPEL2, SEC63, AARS, GLRX2, HSPA9, PPIL4, PFDN2, CWC27, TBCE | 12 | 5.90E-03 |
| nucleolus | DNAJB9, HNF1B, ISY1, MINA, NKAP, PAK1IP1, RCOR3, SNW1, ATF3, ACSS2, BRD4, CCNT1, FXR1, GNL2, HKDC1, KLHL7, LIAS, ME1, NLE1, TSEN15, TRIB3, USP36, ZBTB43, ZNRD1 | 24 | 2.20E-02 |
| transmembrane receptor protein tyrosine kinase activity | EPHA1, EPHA7, EPHB2, FGFR1, FGFR2, ERBB2 | 6 | 2.20E-02 |
| serine family amino acid metabolic process | GCLC, GCLM, PHGDH, PSPH, SHMT2 | 5 | 4.00E-03 |
| Heat shock protein Hsp70 | HSPA4, HSPA9, HSPA13, HSPA5 | 4 | 2.40E-03 |
| protein stabilization | MDM4, SOX4, COG3, TBRG1 | 4 | 3.40E-02 |
| sulfur metabolic process | MTRR, CHST4, GCLC, GCLM, GLRX2, IDH1, LIAS, PHGDH | 8 | 2.80E-02 |
| NADP or NADPH binding | MTRR, DHCR7, ALDH1L2, CYP2R1, FDFT1, FMO5, HSD17B7, IDH1, ME1, SC5DL | 10 | 8.60E-03 |
| oxidoreductase | MTRR, DHCR7, ATP6, COX3, UGDH, ALDH1L2, CYP2R1, FDFT1, FMO5, HSD17B7, IDH1, JMJD1C, LOX, ME1, PHGDH, SCD, SC5DL, SRXN1 | 18 | 8.60E-03 |
| cofactor binding | MTRR, TGDS, UGDH, ALAS1, ASNS, FMO5, GCLC, GPT2, IDH1, ME1, MTO1, PHGDH, SHMT2 | 13 | 1.70E-02 |
| monosaccharide metabolic process | NANP, UGDH, ATF3, CHST4, GFPT1, GSK3B, HKDC1, IRS2, PCK2, PGM3, PIK3CA, PMM2, PPP1R2, SERP1 | 14 | 4.70E-03 |
| gliogenesis | NF1, PHGDH, TSPAN2, TGFB2, ERBB2, ZNF488 | 6 | 2.50E-02 |
| BTB/POZ | PATZ1, ANKFY1, KLHL14, KLHL24, KLHL7, MYNN, ZBTB43, ZBTB47, ZNF295 | 9 | 1.00E-02 |
| Zinc finger, CCCH-type | RBM26, TRMT1, U2AF1, MKRN2, ZC3H15 | 5 | 3.40E-02 |
| heart development | SOX4, SOX6, BMP4, COL4A3BP, CXADR, DLC1, ECE2, MIB1, NF1, OBSL1, TGFB2, TPM1, ERBB2 | 13 | 9.40E-03 |
| organelle envelope | DHCR7, BNIP1, COX16, COX3, ATP6, FAF1, GRPEL2, NLRX1, NXT1, NPC1, ERN1, XPOT, KPNA1, MSTO1, NUPL1, SHMT2, SLC25A33, SCD, TIMM44, TMEM173, TMEM38A, TMEM38B, TNPO1 | 23 | 2.20E-02 |
| regulation of phosphate metabolic process | ADORA1, BMP4, CCNK, CCNT1, DLC1, ERN1, GADD45A, IGFBP3, LRP8, NF1, PAK2, PPP1R2, TGFB2, TRIB3, TNFSF15, TNFRSF10B, ERBB2 | 17 | 1.80E-01 |
| transit peptide | MTRR, COX16, GRPEL2, NLRX1, ALAS1, COQ10B, GLS, GLRX2, HSPA9, LIAS, LIPT1, LONP1, MTO1, PCK2, SHMT2, TIMM44, YRDC | 17 | 7.00E-02 |
