# Supplementary material for: Visualized Gene Network Reveals the Novel Target Transcripts Sox2 and Pax6 of Neuronal Development in Trans-Placental Exposure to Bisphenol A
Source: PLoS One. 2014 Jul 22;9(7):e100576. doi: 10.1371/journal.pone.0100576 (PMC4106758; doi:10.1371/journal.pone.0100576)
Supplement: Information S1 — Description text of microarray data processing and differentially expressed analysis. (DOCX) [file pone.0100576.s003.docx]

Supporting Information S1

SI Materials and Methods

1. Microarray data processing

Two different platform chips (Agilent Whole Human Genome Microarray and Affymetrix HG-U133Plus2.0) were pre-processing using RMA algorithm [[1](#_ENREF_1)] prior to merge each other. We selected genes from all platforms based on the NIH Entrez Gene ID and used the median rank score method with the R package CONOR [[2](#_ENREF_2)] for cross-platform normalization (**Figure S1**). Of 22,277 probes that were common among multiple platforms, 20,184 probes were selected to be in one to one relation between probe and gene. Coefficient of correlation (*r*) in each gene among microarray platforms was measured for considering the differences in microarray platforms. We evaluated the median value of each gene in both platforms and selected the gene sets with high correlation in which the absolute value of the extracting median values between two platforms was less than 1. From the resulting 8,920 genes, we removed genes that were not flagged as “detected” in more than 90% of training data set samples (n=273), considering them to have had either missing or uncertain expression signals. Moreover, all data were normalized per gene in each data set by log_2_ transforming the expression of each gene.

2. Differentially Expressed Analysis

In this study, Limma program was used for differentially expressed analysis in R-based Bioconductor package to calculate the level of differential expression [3]. Briefly, a linear model was fit to the data (with sample means corresponding to the different conditions and a random effect for array), and the list of DEGs with p-value <0.01 were obtained by performing the following comparisons based on high- and low- BPA exposure groups (e.g., low BPA exposure group versus control group, and high BPA exposure group versus control group). After assessment of samples to classes, each gene was assessed for differential expression with Student’s t test. The t tests was conducted both as two-sided for differential expression analysis, and the Q values [4] (estimated false discovery rates) were conducted to account for multiple hypothesis testing.

Following single gene-based significance testing, the expression value of DEGs (p-value < 0.01) clustered the patients for each comparison. DEGs were identified for each comparison to serve as potential gene signature and to classify high- or low- BPA groups. The DEGs were analyzed for enriched biological process terms using the NCBI DAVID server (http://david.abcc.ncifcrf.gov) with default setting [5]. All calculations were carried out under R statistics computing.

**Reference**

1. Lopez-Romero P, Gonzalez MA, Callejas S, Dopazo A, Irizarry RA (2010) Processing of Agilent microRNA array data. BMC Res Notes 3: 18.

2. Rudy J, Valafar F (2011) Empirical comparison of cross-platform normalization methods for gene expression data. BMC Bioinformatics 12: 467.

3. Diboun I, Wernisch L, Orengo CA, Koltzenburg M (2006) Microarray analysis after RNA amplification can detect pronounced differences in gene expression using limma. BMC genomics 7: 252.

4. Storey JD, Tibshirani R (2003) Statistical significance for genomewide studies. Proc. Natl. Acad. Sci. U. S. A. 100: 9440-9445.

5. Huang da W, Sherman BT, Tan Q, Kir J, Liu D, et al. (2007) DAVID Bioinformatics Resources: expanded annotation database and novel algorithms to better extract biology from large gene lists. Nucleic Acids Res 35: W169-175.
